# Multifaceted functional implications of an endogenously expressed tRNA fragment in the vector mosquito *Aedes aegypti*

**DOI:** 10.1371/journal.pntd.0006186

**Published:** 2018-01-24

**Authors:** Matthew W. Eng, Anthony Clemons, Casey Hill, Roberta Engel, David W. Severson, Susanta K. Behura

**Affiliations:** Department of Biological Sciences and Eck institute for Global Health, University of Notre Dame, Notre Dame, Indiana, United States of America; Universita degli Studi di Pavia, ITALY

## Abstract

The mosquito *Aedes aegypti* is the primary vector of human arboviral diseases caused by dengue, chikungunya and Zika viruses. Many studies have shown the potential roles of small RNA molecules such as microRNA, small interfering RNA and PIWI-interacting RNA in vector mosquitoes. The function of tRNA fragments (tRF), the newly discovered class of small RNAs, in mosquitoes is not known. In this study, we show that specific tRFs are expressed in significantly differential manner between males and females of *Ae*. *aegypti* strains. Specific tRFs also show differential response during developmental transition from larvae to adults, as well as after blood feeding of adult females. The expression pattern of tRFs upon blood feeding varied depending upon if the blood contained dengue virus, and also if the females were treated with antibiotic prior to feeding to cleanse of the gut bacteria. Our findings show that a single tRF derived from the precursor sequences of a tRNA-Gly was differentially expressed between males and females, developmental transitions and also upon blood feeding by females of two laboratory strains that vary in midgut susceptibility to dengue virus infection. The multifaceted functional implications of this specific tRF suggest that biogenesis of small regulatory molecules from a tRNA can have wide ranging effects on key aspects of *Ae*. *aegypti* vector biology.

## Author summary

The mosquito *Aedes aegypti* is a major vector of arboviral diseases in subtropics and tropics. The confounding effects of immature development and adult microbiome on the ability of *Ae*. *aegypti* to transmit diseases (vector competence) have gained renewed attention in the recent years. However, the molecular nature of these links/ effects remains unknown. This is major gap in knowledge regarding how vector competence is regulated at molecular level, and how that regulation may be variable among different strains of this mosquito. In this study, we investigated expression of newly discovered class of small RNAs, called tRNA fragments (tRF) in *Ae*. *aegypti* strains. Based on small RNA sequencing and bioinformatics analyses, we show that tRFs are expressed in *Ae*. *aegypti*, and they are associated with significant changes in expression between males and females, during development stages, and post blood feeding responses. A single tRF showed association with sex-biased expression, developmental regulation and in response to blood meals between Moyo-S and Moyo-R strains that differ in midgut susceptibility to dengue virus. The findings of this study are expected to guide future research efforts directed toward examining detailed regulatory mechanisms of tRFs in vector competence of *Ae*. *aegypti* to disease transmission.

## Introduction

Endogenously expressed small regulatory RNAs play diverse biological roles in vector mosquitoes [1–5]. They function in regulating processes that relate to mosquito development, blood digestion, disease vector competence and others [6–10]. More recently, studies have shown that fragments endogenously generated from transfer RNAs, referred to as ‘tRNA-fragment’ (tRF), play active roles in various biological functions in diverse organisms [11–14]. The tRFs are produced from either mature tRNAs or their precursor transcripts, not as random degradation products, but by cleavage at specific sites by specific ribonucleases whose precise mechanisms are not fully understood [15]. While tRFs ranges from 13 to 32 nucleotides (nt) in length, relatively longer fragments (30–35 nt) known as ‘tRNA halves’ are also produced as functional molecules from mature tRNAs under certain conditions [16]. The functions of tRFs are multifaceted: they regulate diverse target genes like microRNAs, have association with human cancer and other diseases, confound cell viability, influence RNA stability and degradation, affect sperm maturation and fertilization, and compromise translational selection of genes [17–21]. In insects, limited research has been performed on tRFs. In *Drosophila*, a role for tRFs in developmental regulation has been shown [22]. In the silk worm, tRFs have been mapped to the D-loop, anticodon and TψC loop of different tRNAs [23]. In the green-bottle blowfly, small RNAs mapping to the 5’ end of mature tRNA Gly-GCC have been reported [24]. Despite the increased research interest on identifying and curating tRNA-derived small RNAs in different species [25], no study on tRFs has been reported in mosquitoes.

In this study, we investigated tRF abundance in a genome-wide manner in the mosquito *Aedes aegypti* that acts as the primary global vector of different arboviral diseases of humans [26–29]. The primary objective of this study is to determine if tRFs are expressed and differentially regulated in *Ae*. *aegypti*. Towards achieving that broad objective, we profiled tRF expression in different biological samples that varied in sex, developmental stage and treatments (such as antibiotic, blood feeding or oral challenge of dengue virus). Furthermore, we generated these biological samples from two laboratory strains of *Ae*. *aegypti*, Moyo-S and Moyo-R (see Methods), in order to compare tRF expression between the strains for different biological samples. In addition, we generated tRF expression profiles in males and females of additional three strains to investigate if tRF regulation is sex-biased among the strains. The results of this study show that different tRFs accumulate in significantly differential abundance at different developmental stages, between sexes, and in response to microbiome perturbation by antibiotic treatment. The study further shows that tRF profiles are altered during post-feeding times with a naïve or dengue virus infected blood meal. Importantly, our data further reveal that a specific tRF is commonly differentially expressed between males vs. females, during development as well as has association with the microbiome and dengue virus infection, suggesting its potential multifaceted functional role in *Ae*. *aegypti*.

## Materials and methods

### Ethics statement

This study was performed in accordance with the recommendations in the Guide for the Care and Use of Laboratory Animals of the National Institutes of Health. The animal use protocol was approved by the University of Notre Dame Institutional Animal Care and Use Committee (Study # 11–036).

### Experiments and mosquito strains

**Fig 1:**
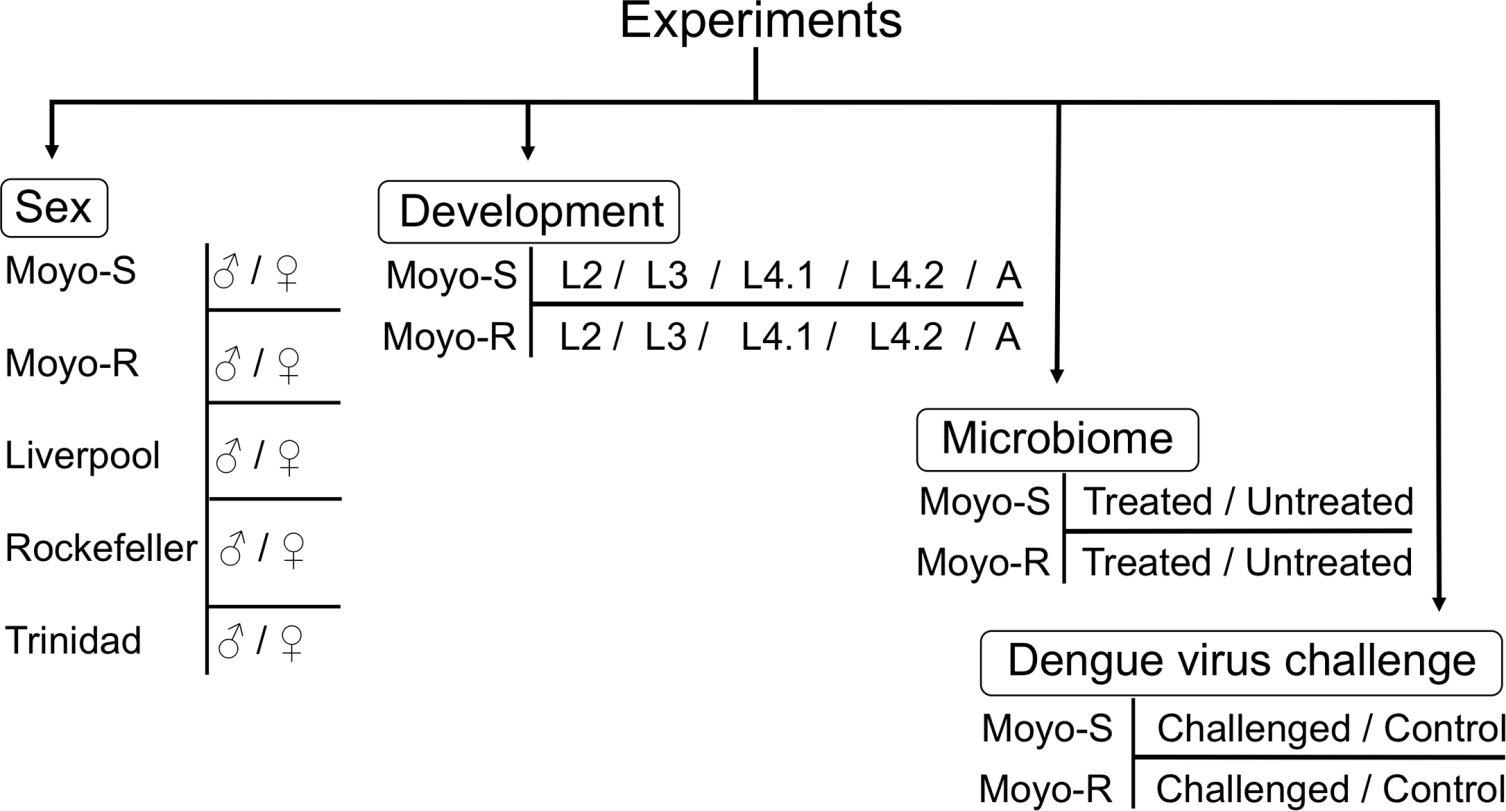
Summary of experiments conducted in this study. It shows the description of four experiments conducted to compare tRF expression profiles in samples representing differences in sex, development, microbiome and dengue virus challenge. The mosquito strains and the samples analyzed in each experiment are shown. The abbreviation of samples used for developmental stages are L2: 2^nd^ instar larvae, L3: 3^rd^ instar larvae, L4.1: 4^th^ instar larvae day1, L4.2: 4^th^ instar larvae day2, and A: adults.

In this study, four experiments were conducted (Fig 1). These experiments were designed to study relevance of tRF expression to sex, development, microbiome and vector response to dengue virus challenge in *Ae*. *aegypti*. The two laboratory strains Moyo-S and Moyo-R were used in all the four experiments. They are sub-strains of Moyo-In-Dry strain that were originally selected for differential susceptibility to the avian malaria parasite, *Plasmodium gallinaceum* [30], and subsequently determined to show high and low susceptibility to dengue virus respectively [31–32]. To compare tRF expression in males vs. females in *Ae*. *aegypti*, we included three additional strains (Liverpool, Rockefeller and Trinidad) along with Moyo-S and Moyo-R. The mosquitoes were reared and maintained in environmental chambers following standard conditions [33]. Environmental chambers were held at 28°C, 80% relative humidity, with a 16 h light: 8 h dark cycle, which included a 1-h crepuscular period to simulate dawn and dusk.

### Profiling tRF expression in males vs. females

Unlike the other three experiments where multiple samples representing different developmental stages or treatments were used, this experiment had only two biological samples per sex if Moyo-S and Moyo-R were used alone. So, we included the three additional strains (Liverpool, Rockefeller and Trinidad) in order to obtain five biological samples per sex. The Liverpool and Rockefeller strains are long-standing and widely used laboratory strains of *Ae*. *aegypti* in the research community. Furthermore, the reference genome sequence of *Ae*. *aegypti* was assembled from a highly inbreed sub-strain of the Liverpool strain [34]. The Trinidad strain was initiated from eggs collected in Trinidad, West Indies in 2012. All the strains were maintained at the University of Notre Dame at the time of conducting this study. Upon adult emergence, equal number of males and females (n = 6) from each strain were pooled for library construction and sequencing.

### Profiling tRFs for developmental stages

The abundance of tRFs in second, third, fourth instar larvae and adults (3-days post-emergence, mixed sex) of Moyo-S and Moyo-R strains were profiled. For the fourth instar larvae, samples were included from two time points, day 1 and day 2, the day 2 time being closest to the onset of pupation. Two biological replicates per developmental stage per strain were included in library construction.

### Blood feeding experiments

We conducted two independent experiments to study how tRFs are expressed in Moyo-S and Moyo-R females after blood feeding. In the 1st experiment, we fed Moyo-S and Moyo-R females with either non-infectious blood meal (naïve blood) or blood mixed with dengue virus serotype-2 strain JAM1409 (infectious blood) and profiled tRF expression at 24 hours and 48 hours post feeding. For mosquito feeding, equal volumes of defibrinated sheep blood (Colorado Serum Company, CO, USA) mixed with either uninfected C6/36 cell suspension or dengue virus infected C6/36 cell suspension were used. The freshly made blood meals were orally fed to 3-day old adult females of the two strains. Blood/cell suspensions were warmed to 37°C and aliquoted into glass artificial membrane feeders whose openings were covered with sausage casing. Female mosquitoes were allowed to feed for ~20 minutes. Fully engorged females were separated and maintained at 28°C in 80% relative humidity and provided 5% sucrose solution. Groups of mosquitoes were removed at day1 (24 hr post feeding) and day2 (48 hr post feeding), and frozen at -80°C until extraction of RNA. Five blood fed mosquitoes from each strain and time point were used for RNA isolation. For sequencing, we used two biological replicates for each post-feeding day per strain per treatment. Thus, total of 16 libraries [2 strains x 2 treatments (naïve vs DENV) x 2 post-feeding time x 2 biological replicates] were sequenced for this experiment.

In the 2nd experiment, we wanted to profile tRF expression upon blood feeding (3 hours) in females that were cleansed for midgut bacteria relative to control (fed without cleansing). For this experiment, the Moyo-S and Moyo-R pupae were separated into two cages prior to adult emergence. The subsequent steps were followed as described elsewhere [35]. Briefly, after adult emergence, the two cages were provided with different sugar solution treatments. One cage was provided a control sterilized 8% sugar solution, while the other cage was provided an 8% sterilized sugar solution containing 2% penicillin-streptomycin and 0.8% gentamicin sulfate for bacterial cleansing [36]. Both sugar treatments were provided *ad libitum* using saturated sterilized cotton balls. Mosquitoes were allowed 8 days of sugar feeding to assure optimal clearance of midgut bacterial populations among the antibiotic treated samples. To verify bacterial clearance, we followed methods described earlier [35]. The midguts were dissected from 20 random females for each treatment and homogenized in sterilized PBS. Thereafter, 1.5 μl aliquots of the midgut solutions were prepared and spread on blood agar plates under sterile conditions. After 3 days, plates were examined for microbial growth. Bacterial clearance was also determined using a culture-independent method utilizing 16S rRNA amplification [35]. The antibiotic treated and untreated females were then starved for 24 hours and then separately provided artificial blood meal prepared using defibrinated sheep blood as described above. Females were allowed ~20 min to feed to engorgement and fully engorged females were isolated in separate cups and maintained at 28°C in 80% relative humidity and provided 5% sucrose solution. Midguts were collected at 3 hr post blood feeding from both groups (the blood was removed via dissection), and stored in RNA*later* at -80°C. Three independent biological replicates, each consisting of a pool of midguts from 20 blood fed mosquitoes, were used for library construction.

### Small RNA sequencing

Total RNA was extracted using Qiagen RNeasy mini kit according to manufacturer’s protocol. Quality of RNA was assessed using a Bioanalyzer (Agilent, Santa Clara, CA, USA) according to manufacturer’s protocol. Quantity of RNA was determined using Qubit Fluorometer (Life Technologies, Grand Island, NY, USA) according to manufacturer’s specifications. Library preparation was conducted using the Epicentre ScriptMiner kit (Cat. No. SMMP101212) following the suppliers protocol. The sequencing was performed using Illumina MiSeq at the Genomics Core Facility at the University of Notre Dame.

### Mapping of tRNA fragments

**Fig 2:**
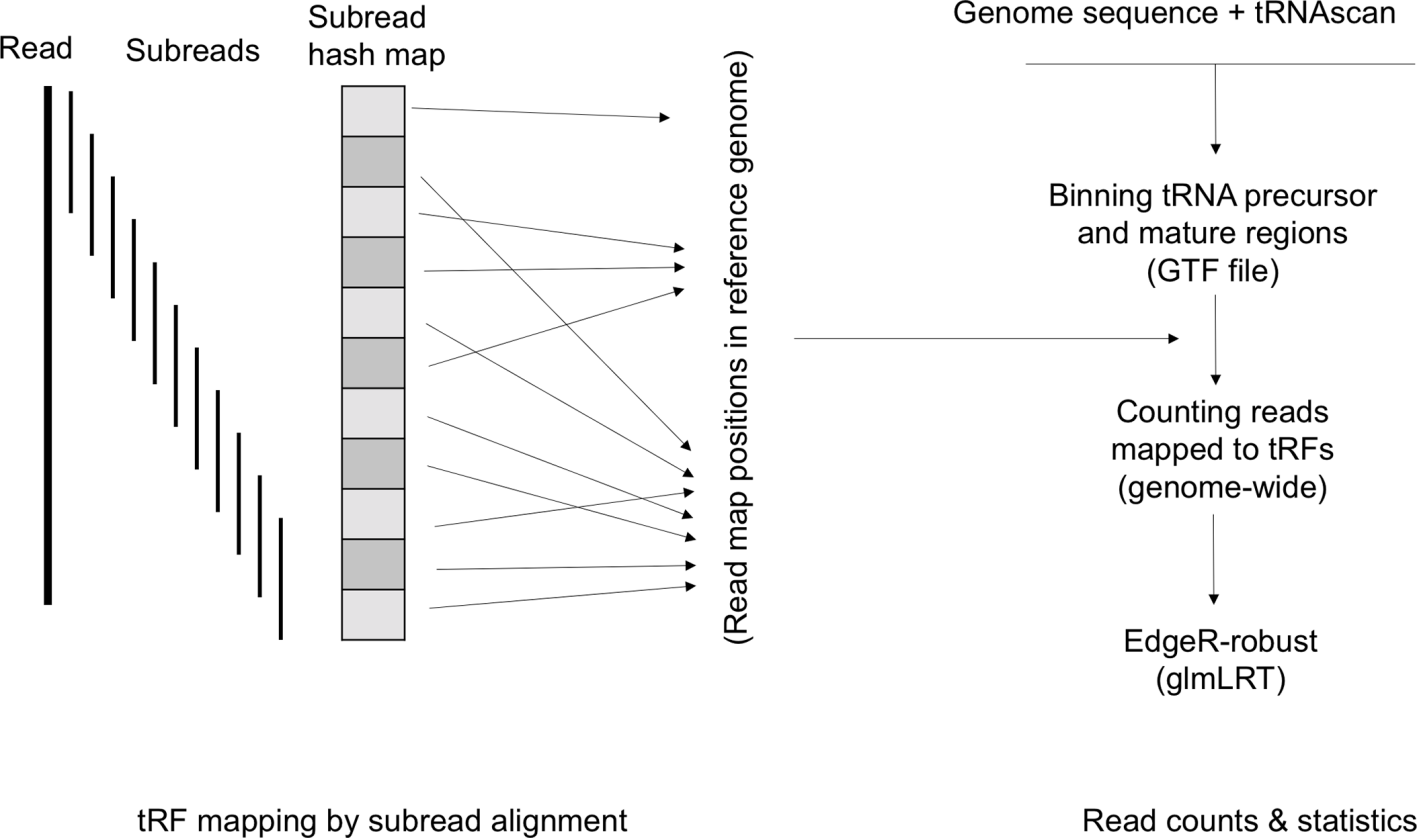
Analysis of small RNA sequences for tRF mapping and quantification. In the first step, the sequence reads after removing adapters and quality trimming were aligned to the full index of reference genome AaegL3 using Subread aligner. In the second step, tRNA genes were annotated from the genome sequences using tRNAscan tool. The sequences of mature and flanking regions of mature tRNA sequences were binned and the genomic coordinates of resulting bins were used as a general transfer format (GTF) file along with the alignment files generated from the first step to count reads. The read counting was done using the StringTie tool. The changes in tRF abundance was analyzed from the read count data of tRFs using the edgeR robust algorithm.

To identify differentially expressed tRFs from the sequence data, we employed a method as illustrated in Fig 2. Unlike the indexing methods of reference genome in RNA-seq analysis, this method employs a full index reference mapping strategy [37]. In this method, first we generated a full index of the AaegL3 reference assembly by comprehensively extracting 16-mers from every position of the genome. The indexed reference genome was then used for mapping of small RNA sequence reads using Subread aligner [37] that determines map position of the sequence reads in the reference genome (AaegL3) using a seed-and-vote strategy [37]. The threshold value of 4 (the number of consensus subreads required for reporting a hit) was used for mapping reads across all the samples. The positions of tRFs in the reference genome were determined from the genomic coordinates of parental tRNAs, and the location of fragments within the tRNAs. These subsequences of tRNAs corresponded to different loop and arm regions of secondary structures of tRNAs as predicted by tRNAscan [38]. The information of genomic positions of tRFs was then used to quantify the number of reads mapped to each tRF in a genome-wide manner by StringTie [39]. To compare tRF expression between different sample groups, we used the R package edgeR-robust [40] which normalizes the read count data by using trimmed mean of M-values method (TMM), and performs statistical inference of differential expression of tRFs by fitting the normalized data to weight-based generalized linear model (glm) and conducting a likelihood ratio test of observed weights.

### Statistical analysis

All statistical analyses were performed in *R*. To analyze tRF expression in males vs. females of different strains, we implemented an information theory approach based on mutual information (MI). MI a measure of the information content that two variables share: a numerical value ranging from 0 to 1 depending on, intuitively, how much knowing one variable would predict variability of the other. In this approach, the mutual information (MI) of tRF expression variation was determined in pair-wise manner across males and females of the strains using R package *minet* [41]. The MIs were calculated using Spearman estimator, with no discretization method applied to the data prior to calculation.

## Results

### Small RNA sequencing and profiling of tRNA fragments

**Fig 3:**
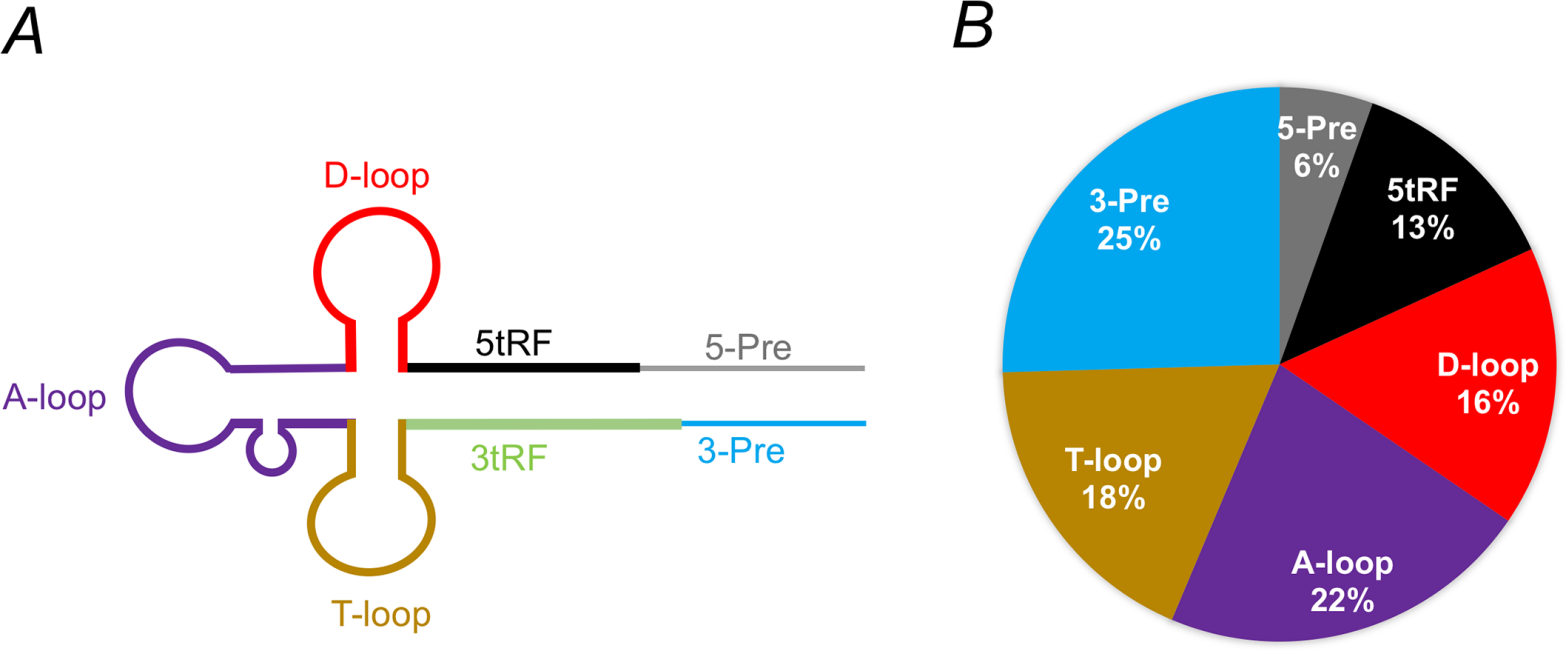
Schematic representation of tRFs and their observed abundances in *Ae*. *aegypti*. **A**) A typical clover leaf secondary structure of tRNAs. The nomenclature of tRFs representing different stem and loop regions (color coded) are shown. The loop names shown represent the loops that contain the base dihydrouridine (D-loop), anticodon triplet (A-loop) and the TΨC sequence (T-loop) where Ψ is pseudouridine. **B**) Pie chart showing the proportion of abundance of tRFs identified in all samples.

We performed Illumina (MiSeq) sequencing of small RNA libraries (n = 40) representing different biological samples (S1 Table). All the sequences generated in this study have been deposited at the Gene Expression Omnibus database (https://www.ncbi.nlm.nih.gov/geo/) under the accession number GSE101956. The MiSeq sequencing generated ~ 1.98 million small RNA reads per sample. This estimate is based on the average number of reads generated from sequencing of the 40 samples (S1 Table). A total of 79,309,784 reads were generated across all the samples. The number of reads generated from different sample groups is listed in Table 1. The raw reads were processed for adapter removal by cutadapat (https://github.com/marcelm/cutadapt) and quality trimming by sickle (https://github.com/najoshi/sickle), and then mapped to the reference genome AaegL3 for quantification of tRNA fragments using a method illustrated in Fig 2. The binary alignment map (bam) files generated from alignments were used to identify reads that mapped to different parts of tRNAs. We followed the tRF nomenclature as described earlier [14] which classifies tRFs originating from either the 5’- or the 3’-end of precursor sequences of tRNA genes (5-Pre and 3-Pre respectively) as well as fragments originating from either the 5’- or the 3’-end of mature tRNAs (5tRF and 3tRF respectively). We extended this nomenclature to further include tRFs that might be originating from different tRNA loops [12]. To achieve this, first we predicted different regions within clover-leaf secondary structure of tRNAs using tRNAscan [38] from sequences of all the tRNA genes (n = 984) as predicted in AaegL3.1 annotation (www.vectorbase.org). We found that 109 tRNAs are possibly either pseudogenes tRNAs or tRNAs for non-standard amino acids SeC(e) or tRNAs that contain intron sequences in the precursors (Tyr, Ile and Leu tRNAs). These tRNAs were excluded from further study for the sake of simplicity of analysis. Based on the secondary structures predicted by tRNAscan, a total number of 6,118 bins from 875 tRNAs were generated from both mature and flanking sequences. The sequence reads that mapped to different parts of tRNAs were quantified using the StringTie tool [39]. Using this mapping and read quantification approach, we observed that only a total of 55 tRFs are ‘expressed’ in *Ae*. *aegypti* (S2 Table). We defined ‘expressed’ tRFs as those fragments that showed more than 100 reads mapped in at least 3 samples. Interestingly, each of these tRFs originated from unique tRNA genes; no tRNA produced more than one tRF. Moreover, the tRFs showed a strong bias in their origins within tRNAs. The tRFs mapping to the 3’-end precursor (3-Pre) sequences of tRNAs accounted the most, while no tRF originating from the 3’-end of mature tRNA sequences (3tRFs) was detected in any sample (Fig 3). Of all the tRFs we identified, one derived from the 3-Pre region of a Gly-GCC tRNA (VectorBase gene # AAEL016015) was the most abundantly expressed tRF in *Ae*. *aegypti* across all samples (S2 Table). The expression level of tRFs varied among samples suggesting differential regulation of tRFs. The expression pattern of tRNA fragments further suggested that they were generated from specific tRNA genes and specific sites within tRNAs, and hence they were not random cleavage products of tRNAs.

**Table 1:** Description of samples investigated in this study. Abbreviations used in sample names are- MS: Moyo-S, MR: Moyo-R, and DENV: dengue virus.

Sample group	Description	No. of reads
MR-antibiotic treated	Antibiotic treatment- Moyo-R strain	3293734
MR-antibiotic untreated	No antibiotic treatment- Moyo-R strain	3565178
MS-antibiotic treated	Antibiotic treatment- Moyo-S strain	3816752
MS-antibiotic untreated	No antibiotic treatment- Moyo-S strain	3618226
MR-DENV	DENV challenge- Moyo-R strain	8546216
MR-Control	Control: No DENV challenge- Moyo-R strain	10756770
MS-DENV	DENV challenge- Moyo-S strain	8281386
MS-Control	Control: No DENV challenge- Moyo-S strain	8783758
MR-immature	Immature larvae (stages 2,3 and 4)—Moyo-R strain	4219050
MR-adults	Moyo-R adults (mixed sex)	802882
MS-immature	Immature larvae (stages 2,3 and 4)—Moyo-S strain	4251764
MS-Adults	Moyo-S adults (mixed sex)	931800
Males	Males from 5 strains	9574404
Females	Females from 5 strains	8867864

### Sex-biased expression of tRFs in *Ae*. *aegypti*

**Fig 4:**
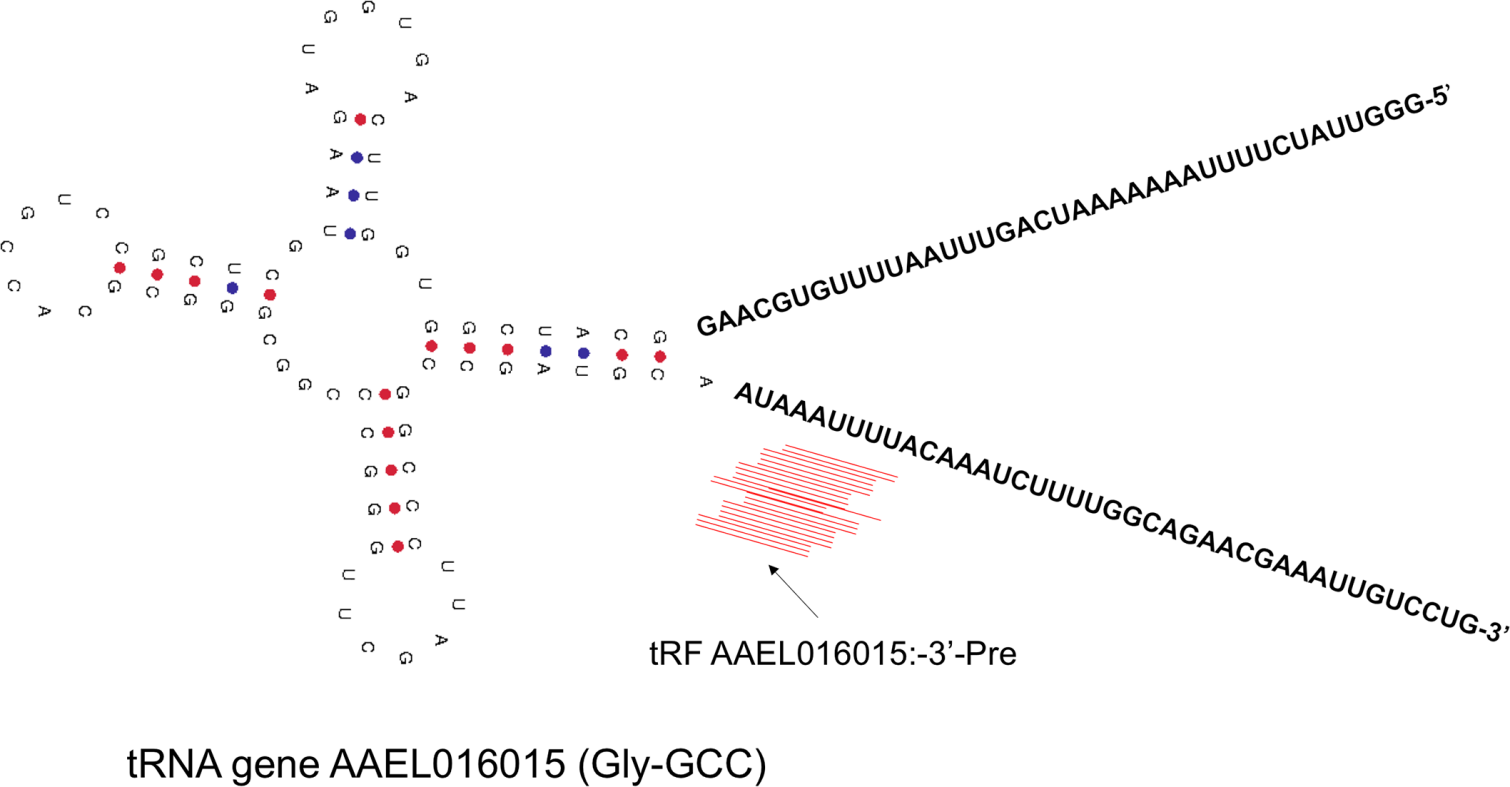
The tRF (AAEL016015: 3’-Pre) whose expression varied in sex-biased manner across the strains of *Ae*. *aegypti*. The mature tRNA region in the gene sequences of AAEL016015 along with 5’-end (blue) and 3’-end (red) flanking precursor sequences are shown. Immediately flanking the 3’end of the mature tRNA (representing 3’ precursor of the tRNA), mapped sequence reads were identified that represented the tRF AAEL016015: 3’-Pre. The secondary structure of the mature tRNA with anticodon GCC is shown.

We profiled tRF expression in males and females of different (n = 5) laboratory strains of *Ae*. *aegypti*. They included Liverpool (Lvp), Rockefeller (Rock), Moyo-S, Moyo-R and Trinidad (Trini). By profiling these 10 samples, we identified a total of 31 tRFs that showed biased abundance in males *versus* females across strains based on principal component analysis (S1A Fig). To determine the level of sex-biased expression of tRFs between strains, we performed pair-wise mutual information (MI) analysis of tRF expression of males and females between strains. MI is a measure of the mutual dependence between the two variables that infers how much one variable tells us about another variable. From this analysis, we observed that individual strains had varying pattern of sex-biased changes in expression of tRFs (S1B Fig). The expression of tRFs revealed differential cluster patterns between males and females as shown in a tanglegram generated from hierarchical clustering (S2 Fig). We identified 4 tRFs that showed consistently higher expression in females compared to males across the strains (female biased expression), and 12 tRFs that had higher expression in males compared to females (male biased expression) (Table 2). Fitting the expression data of the 31 tRFs into a weighted generalized linear model implemented in edgeR robust^48^, we identified three tRFs that showed significantly differential expression (*p* < 0.05) between males and females. Among these, a 3-Pre tRF produced from the tRNA gene AAEL016015 (Gly-GCC) (Fig 4) showed female-biased expression across all the five strains. On average, its mean read count was 17334.6 in females and 10792.4 in males. On the other hand, two 3-Pre tRFs produced from tRNA-Ala genes (AAEL016845 and AAEL016846) revealed male biased expression that had 4.5 to 4.6 thousand mean read counts in males compared to 2.3 to 2.4 thousand reads in females.

**Table 2:** List of tRFs showing sex-biased expression in *Ae*. *aegypti*. F: Female, M = Male.

tRNA Gene	tRNA type	tRF class	Mean read counts (F)	Mean read counts (M)	Sex-biased pattern
AAEL016015	Gly-GCC	3-Pre	17334.6	10792.4	Female biased
AAEL016012	Asp-GTC	3-Pre	481.4	309.4	Female biased
AAEL016867	Ala-AGC	5-Pre	427.4	148.8	Female biased
AAEL016866	Lys-TTT	5-Pre	195.2	61	Female biased
AAEL016846	Ala-AGC	3-Pre	2454	4642.6	Male biased
AAEL016845	Ala-AGC	3-Pre	2387.8	4531.8	Male biased
AAEL016779	Thr-TGT	A-loop	526.4	634.8	Male biased
AAEL016742	Ala-AGC	T-loop	159.2	432.8	Male biased
AAEL016064	Pro-CGG	5tRF	246	329.8	Male biased
AAEL016743	Trp-CCA	T-loop	156	419.6	Male biased
AAEL016534	Asp-GTC	3-Pre	118.8	237.4	Male biased
AAEL016533	Gly-GCC	3-Pre	114.8	225	Male biased
AAEL016390	Glu-CTC	5-Pre	114.6	214.2	Male biased
AAEL016947	Gln-CTG	T-loop	127	162	Male biased
AAEL016948	Ala-AGC	T-loop	120.2	159	Male biased
AAEL016542	Phe-GAA	3-Pre	45.8	126	Male biased

### Developmental regulation of tRFs

**Fig 5:**
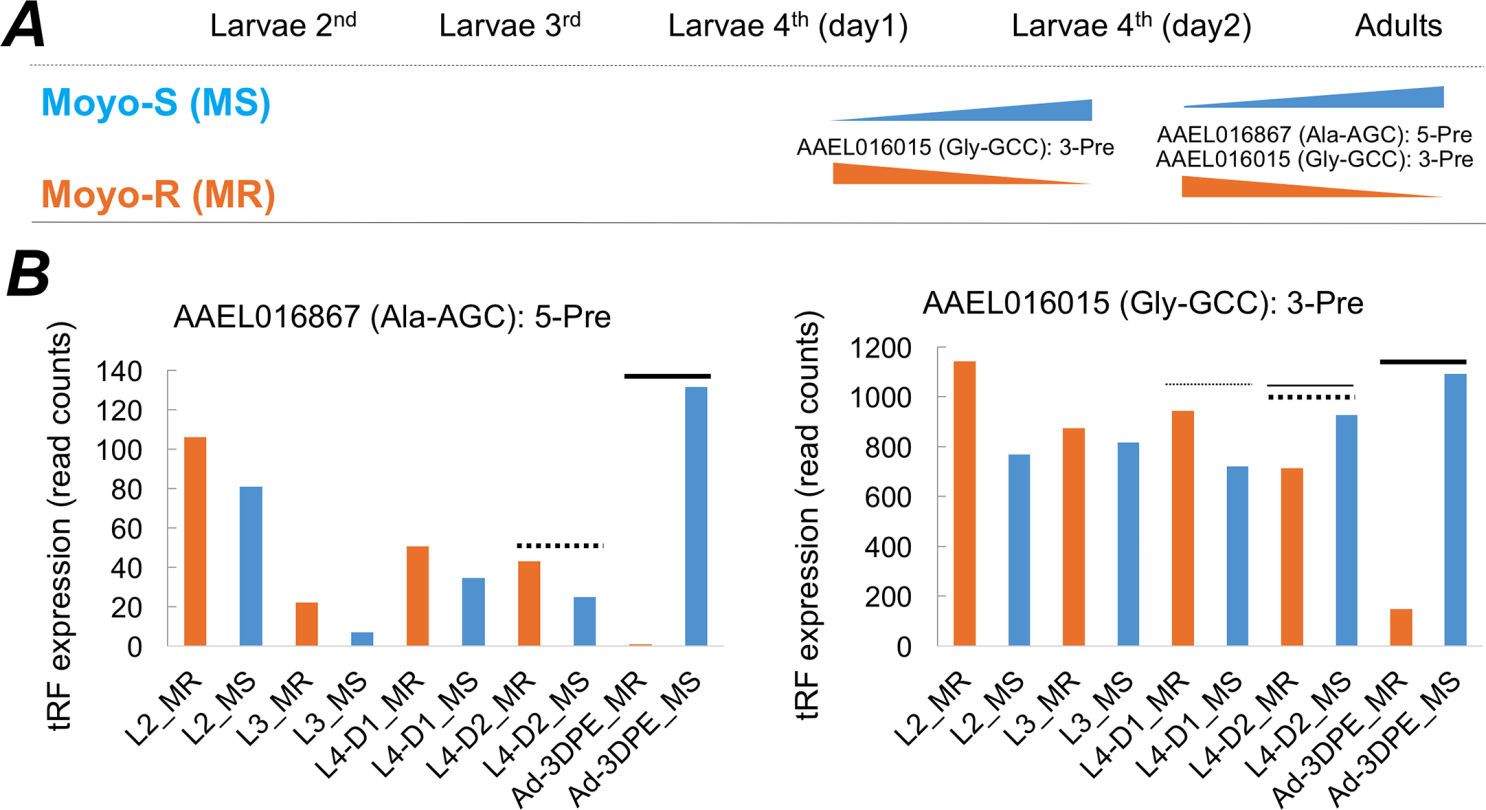
Differential response of tRFs during larval to adult transition. **A**) Two tRFs showed significant (*p* < 0.05) differential expression at specific developmental stages between Moyo-S and Moyo-R strains. **B**) The column graphs show developmental expression patterns of the two tRFs. The developmental stages in the x-axis are abbreviated as L2 (larvae 2nd instar), L3 (larvae 3rd instar), L4-D1 (larvae 4th instar day1), L4-D2 (larvae 4th instar day2), Ad-3DPE (adults 3 days’ post emergence) and the two strains are abbreviated as MS (Moyo-S) and MR (Moyo-R). The dotted vs. solid horizontal black lines over specific columns specifies developmental stages where tRF expression changes were significant (comparisons between thin dotted vs. thin solid, and thick dotted vs. thick solid).

We studied tRF expression at different developmental stages of *Ae*. *aegypti* strains Moyo-S and Moyo-R. We profiled 2^nd^ instar, 3^rd^ instar, 4^th^ instar larvae, and adults (mixed sex) to identify tRFs that are differentially expressed between different developmental stages. The reason for using mixed-sex adults was to account for any sex-biased expression of tRFs in larvae which were not sexed. We compared changes in tRF expression between stages of larvae 2 vs. larvae 3, larvae 3 vs. larvae 4 day1, larvae 4 day 1 vs. larvae 4 day 2, and larvae 4 day 2 vs. adults of the two strains. From this experiment, we observed two tRFs with significant differential expression at specific developmental transition periods. One of them was the female-biased tRF described above (AAEL016015: 3-Pre, Table 2) that showed developmentally regulated expression pattern between Moyo-S and Moyo-R (Fig 5A). In the 4^th^ instar larval stage (between day1 and day2), this tRF showed significant (*p* = 0.034, Fisher exact test) changes in expression between the two strains. It increased in abundance in Moyo-S but decreased in Moyo-R between day1 and day2 within the 4^th^ instar larval stage (Fig 5B). As larvae transitioned to adulthood, the same tRF also showed significant differential expression (*p* = 0.015) in which its abundance diminished almost 5-fold in adults compared to day2 of 4^th^ instar larval stage in Moyo-R, but not in Moyo-S strain. Besides tRF AAEL016015: 3-Pre, we also observed another female-biased tRF that originated from the 5’ precursor region of tRNA AAEL016867 (Ala-AGC: 5-Pre, Table 2) showing significant difference (*p* = 0.007) in abundance in adult mosquitoes compared to larvae (4^th^ instar day 2) between the two strains (Fig 5B). These results suggested that expression of these two tRFs is sex-biased as well as developmentally regulated, and that these tRFs may be associated with sexual dimorphic regulation of adult development in *Ae*. *aegypti*.

### Post blood feeding changes in tRF abundance

**Fig 6:**
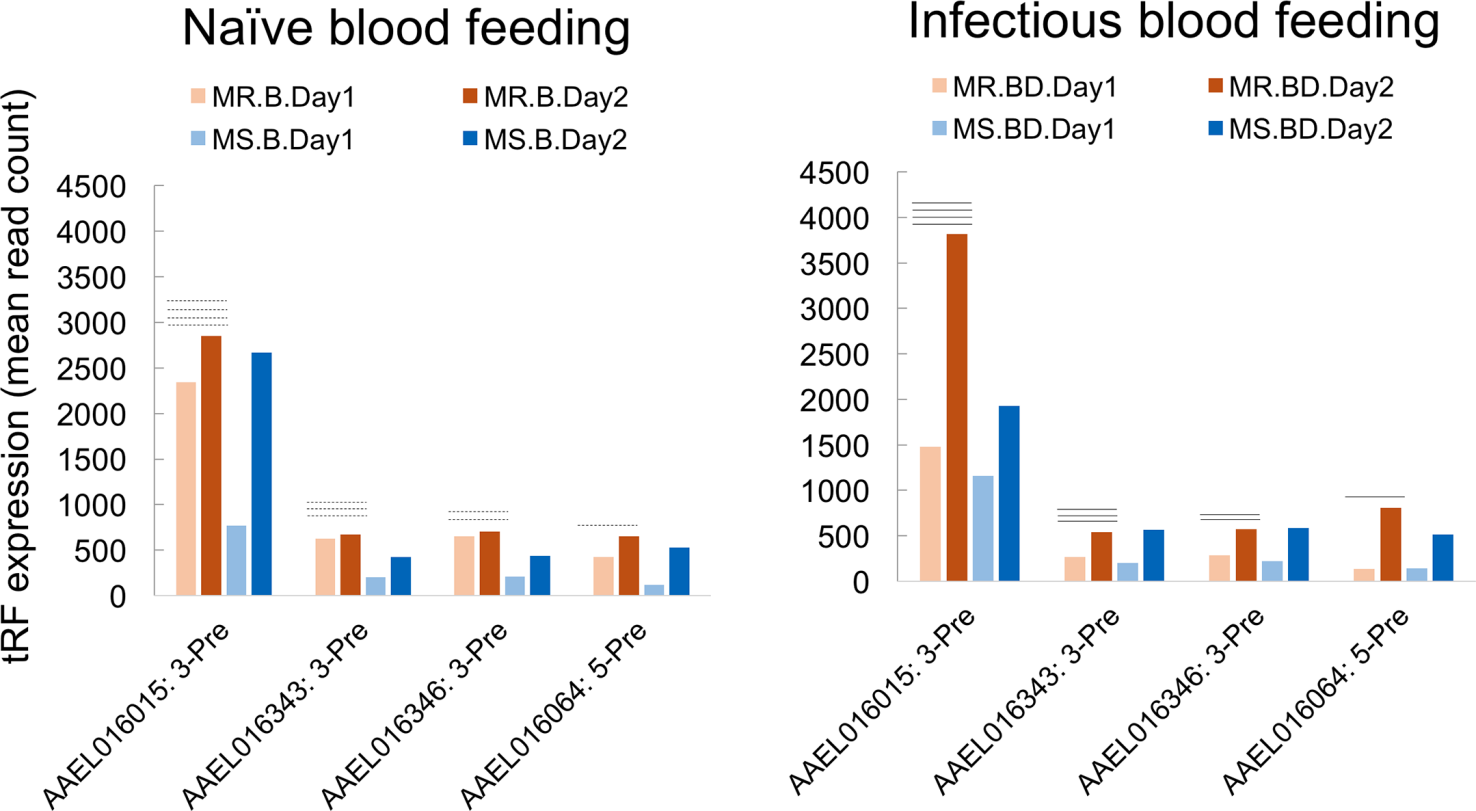
Differential abundance of four tRFs that show differential expression after feeding with dengue virus mixed blood meal compared to naïve blood meal. The column graphs show expression of the 4 tRFs (left: naïve blood feeding, and right: infectious blood feeding). The columns are color coded as light and dark orange for day1 and day2 post feeding in Moyo-R females, and as light blue and dark blue for day1 and day2 post feeding responses in Moyo-S females. The feeding type is shown as either B (blood only) or BD (blood + dengue virus) in the sample legends below the graphs. The dotted and solid horizontal line(s) above column pairs show that presence of dengue virus in blood meal significantly (*p* < 0.05) changes expression of these tRFs in Moyo-R strain only.

We further studied tRF expression in post blood feeding responses in Moyo-S and Moyo-R females. We fed adult females with either naïve blood or blood mixed with dengue virus, and then profiled them for tRF expression on day1 and day2 post feeding. From this experiment, we identified 4 tRFs that showed differential expression between day1 and day2 of naïve blood fed and dengue mixed blood fed mosquitoes of Moyo-S and Moyo-R strains (Fig 6). We observed that the expression of these tRFs changed significantly (*p* < 0.05, Fisher Exact test) upon infectious blood feeding relative to naïve blood feeding between the two strains. Upon naïve blood feeding, these tRFs in Moyo-R females showed little or no changes in expression at the two post feeding times. But, upon feeding with dengue virus mixed blood, they showed elevated expression at day2 relative to day1 post feeding in Moyo-R. In Moyo-S females, however, naïve and infectious blood feeding showed no significant change in expression of these tRFs at the two post feeding time points. These results showed that the presence of dengue virus in the blood was able to alter the expression of tRFs in a strain-specific manner. Interestingly, one of these tRFs was the same AAEL016015: 3-Pre whose expression was female-biased and developmentally regulated as described above. As Moyo-R reflects greater refractory responses to dengue virus [31–32], we speculate that such significant changes in tRFs in Moyo-R may be linked to tRF-mediated regulation of mosquito vector competence to dengue virus infection.

**Fig 7:**
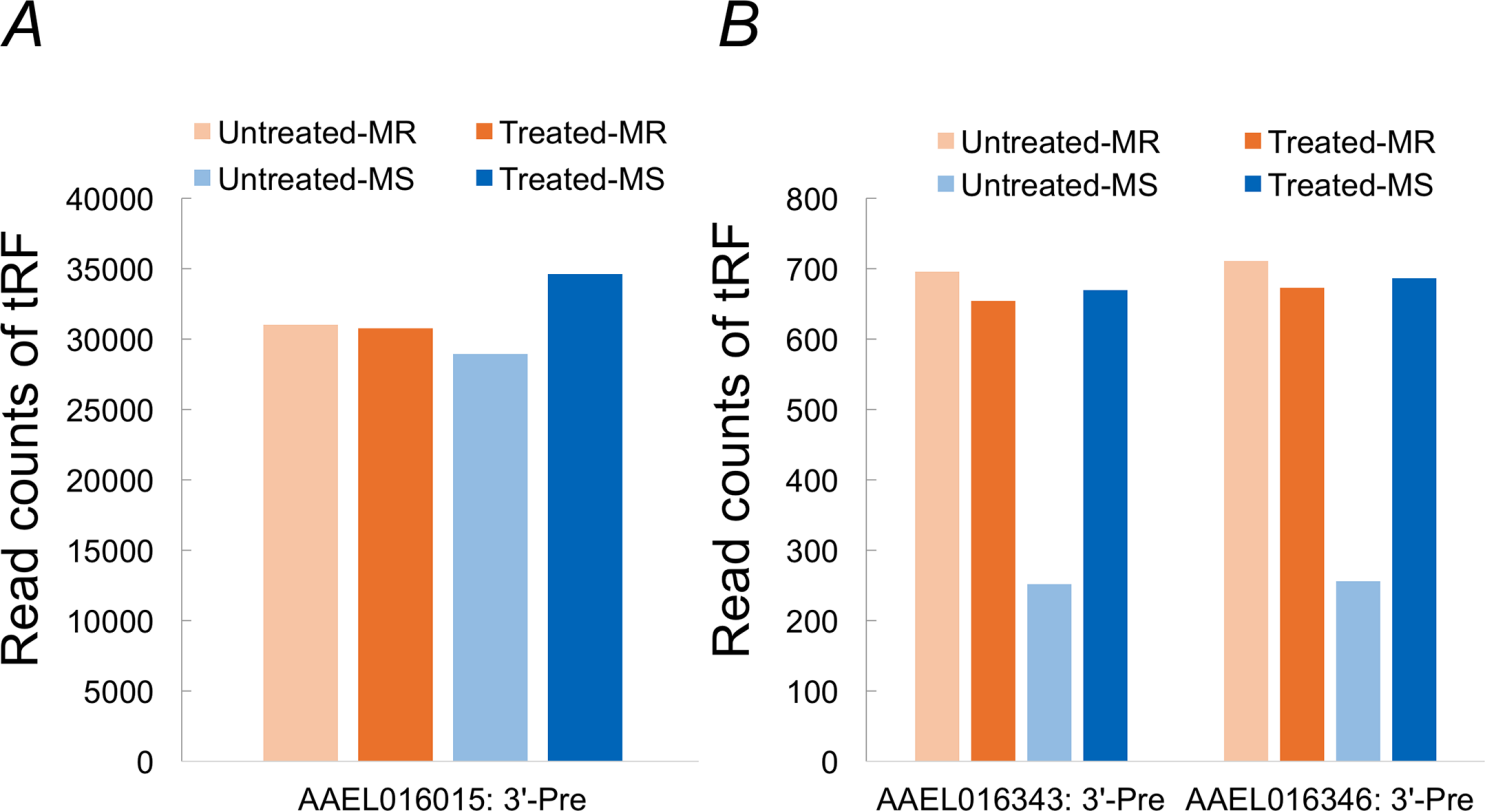
Identification of three tRFs that show differential response to blood feeding after antibiotic treatment of female mosquitoes. **A**) Expression of the tRF AAEL016015: 3’-Pre that shows elevated expression (read counts in y-axis) at 3h post blood feeding in treated females relative to untreated females of Moyo-S strain (MS). But no significant changes were observed in Moyo-R (MR) females. **B**) Moyo-S specific up-regulation of two additional tRFs in treated females. These tRFs are lowly abundant as compared to tRF AAEL016015: 3’-Pre.

We further investigated effect of midgut bacteria of *Ae*. *aegypti* in tRF expression in response to blood feeding. Using antibiotic treatments, we cleansed adult females of Moyo-S and Moyo-R strains separately (see Methods), and compared tRF expression profiles of their midgut with that of non-cleansed females at 3 hr following a naïve blood meal. The results of this experiment showed that bacterial cleaning has a significant effect on differential expression of specific tRFs between Moyo-S and Moyo-R females to blood feeding (Fig 7). Three tRFs showed significantly higher expression (*p* < 0.05, Fisher Exact test) in midgut of cleansed females relative to uncleansed females of Moyo-S strain. However, the changes in Moyo-R females were non-significant. Most interestingly, the tRF AAEL016015: 3-Pre, whose expression was sex-biased, developmentally regulated and responsive to infectious (dengue virus) blood feeding, was also responsive in the midguts of Moyo-S females that were cleansed of bacteria by antibiotic treatment (Fig 7). Together, these results suggested that expression of the tRF AAEL016015: 3-Pre have wide ranging biological effects in *Ae*. *aegypti*.

## Discussion

In this study, we identified fragments of tRNAs that show highly regulated biogenesis in different biological samples of *Ae*. *aegypti*. Biogenesis of active fragments from tRNAs is accomplished by precise cleavage at specific sites within specific tRNAs [15]. Active fragments of tRNAs include both ‘tRNA halves’ which are 30–35 nt in length and tRFs which are shorter than tRNA halves and range from 13 to 32 nt in length [42]. In the current study, we have focused only on tRFs. Based on cleavage sites of tRNAs, tRFs are classified according to their origin within mature or precursor regions, and named accordingly, such as 5tRFs, 3tRFs, 3-Pre, and 5-Pre. However, the nomenclature of tRFs is still a contentious issue [43]. It is unknown if there are universal sites that release the same types of tRFs across species [44]. RNase P and RNase Z are well known enzymes involved in tRNA cleavage [45–46]. Dicer and Angiogenin enzymes are also known to cleave mature tRNAs in some cases [15]. However, there is no Angiogenin gene in *Ae*. *aegypti*. We don’t know the exact cleavage sites of tRNAs in *Ae*. *aegypti*. Given these unknowns, we used a binning approach to quantify tRFs based on mapping of sequence reads to different parts within tRNAs.

We generated 21 nt long reads from sequencing of the small RNA libraries. To optimize bin size of tRNAs for quantifying mapped reads, we conducted a sliding window analysis where bins of varying sizes (15, 20, 25, 30 and 35 bases) of tRNAs were generated and the number of mapped reads to each of these bins in genome-wide manner were analyzed. We found that 15 nt bins produced the highest number of differentially expressed tRFs by edgeR robust than other bins suggesting that a predominant portion of active tRFs present in our samples are likely to be about 15 nt long. Based on this initial analysis, we decided to use 15 nt bins for tRF mapping and for subsequent analyses. We have not attempted to assemble reads to delineate length of individual expressed tRFs. We believe that use of an experimental approach rather than bioinformatics method is a better way to identify intact tRFs and their ends. The profile of tRFs that are active at RISC (RNA-induced silencing complex) by adopting the RNA immuno-precipitation (RIP-Seq) method is possibly a suitable approach for that aim. Previously, this method has been successfully used to detect active tRFs in the silk worm [23].

Given the results from this study that only specific tRNAs produce expressed fragments, a key question is how and on what basis specific tRNAs are selected for biogenesis of tRFs. At ribosome sites during active protein synthesis, mature tRNAs function by recognizing the codon sequences in the mRNA by their anticodon sequences to add the cognate amino acids to the growing peptide chain. Though variation in anticodon sequences of tRNAs produce different isoacceptors (tRNAs cognate to the same amino acid but with different anticodon sequences), sequence variation also occurs in other loops and arms of tRNAs [47]. We have shown in an earlier study that a significant correlation (*p* <0.05) was observed between sequence diversity (π—values) of the anticodon arm and the A-box (internal promoter) of tRNAs in mosquitoes [47]. In addition, sequence variation in the B-box promoter was also significantly correlated (*p* <0.05) with a triplet (63–65) just downstream of this promoter and a part of the TψC arm. We have further shown the existence of a significant association (*p* <0.05) between codon bias and cognate tRNA gene copy numbers in mosquitoes, as well as other insect species [48]. It is thus plausible that genetic variation within tRNAs or translation selection pressure on coding genes (likely the target genes of tRFs) may be associated with selecting specific tRNAs to produce functional tRFs. However, further studies are required to test this hypothesis.

The mapping of small RNA reads to tRNAs in our study showed that many tRNAs had no read mapped. Some tRNAs showed only few reads (< 10 reads) mapping to specific bin(s) in either one or two samples only. As isoacceptors of tRNAs are highly similar in sequences [47–48], we chose the see-and-vote mapping strategy of Subread aligner for tRF mapping that minimizes spurious hits by relying on alignment score of subreads to determine the final map location of a read [37]. If our mapping approach had resulted spurious hits, we would have observed random ‘hits’ to gene copies of different tRNA isoacceptors. But, we observed very precise mapping results where tRFs are localized with only specific tRNA genes. In fact, majority of the tRNA genes predicted in *Ae*. *aegypti* genome show no read mapping at all. We quantified a total number of 6,118 fragments binned from 875 tRNAs, but only 55 (S2 Table) were detected as expressed in the whole genome. Thus, we claim that the full reference based subread mapping approach is highly reliable and sensitive method of mapping tRNA fragments using small RNA sequencing data. However, currently there is no specific best practice in tRF mapping on how many reads should map in order to qualify a tRF as ‘expressed’. There are guidelines to define minimum threshold of read counts to filter out low-expression genes in RNA-seq analysis, but currently we do not have such a guideline in tRF-seq analysis. We selected 100 as the minimum number as this threshold allowed us to identify tRFs that were expressed with greater than 100 reads in more than 3 samples. However, some tRFs including the AAEL016015: 3’-Pre that we highlight in this report is expressed with thousands of reads (S2 Table). In fact, the AAEL016015: 3’-Pre is the most abundantly produced tRF in *Ae*. *aegypti* across samples. At the same time, its expression appears to be tightly regulated as seen in significant changes in read counts between samples. This included significant changes in abundance between males and females, developmental transition from larval to adult stages, antibiotic cleansing of female gut bacteria and post blood feeding responses. Based on the expression of the tRF (AAEL016015: 3-Pre), we propose that this active tRNA fragment might regulate genes associated with sex, development, gut microbiome as well as genes that respond to blood feeding.

It is believed that a functional tRF binds to messenger RNAs similar to microRNAs binding to target mRNA, causing regulation of target genes at the posttranscriptional stages [49]. In our previous studies, we have predicted miRNAs that target conserved developmental genes in flies and mosquitoes [50], and identified genes that showed significant changes during different developmental stages of *Ae*. *aegypti* [51]. As miRNAs and tRNA fragments may regulate gene expression by similar mechanisms [49], it is likely that tRFs that are differentially expressed during transition from larval to adulthood could target genes key to adult emergence. However, we have not predicted those target genes in this study. The changes in expression of this particular tRF upon infectious (dengue virus) blood feeding suggest its possible role in vector response to dengue virus infection. A similar result was observed in a previous study where differential expression of a single tRF was observed in response to respiratory syncytial virus infection [52]. The results of our current study supports the report on emerging roles of tRFs to viral infections [53]. Furthermore, the multifaceted functional implication of tRF (AAEL016015: 3-Pre) makes it a suitable small RNA regulator for further investigating its possible role in vector-virus interactions and pathogen dissemination to humans by blood feeding of *Ae*. *aegypti*. To conclude, the genome-wide analysis of tRNA fragments in *Aedes aegypti* by small RNA sequencing identified active tRFs in different biological samples. Specific tRFs revealed sex-biased expression in multiple laboratory strains. A single sex-biased tRF was identified that showed association with development and post-blood feeding responses. Thus, we claim that tRFs are active in this mosquito, and may play diverse role in disease vector biology.

## Supporting information

S1 Table Description of samples used in the study. (DOCX)

S2 Table List of 55 expressed tRFs and their mean read counts in males and females. (DOCX)

S1 Fig Sex-biased changes in tRF abundance in *Ae*. *aegypti*. **A**) Principal component analysis of tRF abundance in males and females of 5 laboratory strains. The plot shows that males and females have different abundances of tRFs (the dotted line differentiates the two sexes). **B**) Plot of mutual information (MI) of variation of tRF abundance among males and females of the 5 strains. The pair-wise sample comparisons show MI value equals to 1 on the diagonal and the color code (blue to red) represents decreasing values of MI (MI value ranges from 0 to 1).
(TIF)

S2 Fig Tanglegram of tRF expression in males and females of *Ae*. *aegypti*. The variation of tRF expression in males across five strains is on left and that of females among the same strains are shown on right side of the plot. These expression patterns are shown as dendrograms of hierarchical clustering of tRF expression variation in males *versus* females. For example, the two tRFs AAEL016783_3-Pre and AAEL016015_3-Pre cluster together with AAEL016845_3-Pre, AAEL016846_3-Pre, AAEL016761_A-loop and AAEL016763_A-loop in males but cluster separately from those tRFs in females. The cluster branch and nodes are color coded and lines connecting the nodes between the two clusters indicate cluster position of tRFs in males vs. females. Dotted branches show samples with low cluster distance. The scales on the bottom represent branch lengths which were determined from calculating cluster distance by Ward’s method from expression data.
(TIF)
