# Supplementary material for: Multifaceted functional implications of an endogenously expressed tRNA fragment in the vector mosquito Aedes aegypti
Source: PLoS Negl Trop Dis. 2018 Jan 24;12(1):e0006186. doi: 10.1371/journal.pntd.0006186 (PMC5783352; doi:10.1371/journal.pntd.0006186)
Supplement: S1 Table — (DOCX) [file pntd.0006186.s001.docx]

**S1 Table.** Description of samples used in the study.

| Sample # | Sample name | *Aedes aegypti* strain | Sample description |
| --- | --- | --- | --- |
| Sample 1 | LVP-female | Liverpool | Female |
| Sample 2 | LVP-male | Liverpool | Male |
| Sample 3 | MoyoR-female | Moyo-R | Female |
| Sample 4 | MoyoR-male | Moyo-R | Male |
| Sample 5 | MoyoS-female | Moyo-S | Female |
| Sample 6 | MoyoS-male | Moyo-S | Male |
| Sample 7 | Rock-female | Rockefeller | Female |
| Sample 8 | Rock-male | Rockefeller | Male |
| Sample 9 | Trin-female | Trinidaa | Female |
| Sample 10 | Trin-male | Trinidad | Male |
| Sample 11 | MR-L2 | Moyo-R | Larval instar 2 |
| Sample 12 | MR-L3 | Moyo-R | Larval instar 3 |
| Sample 13 | MR-L4-D1 | Moyo-R | Larval instar 4.day1 |
| Sample 14 | MR-L4-D2 | Moyo-R | Larval instar 4.day2 |
| Sample 15 | MR-Ad-3DPE | Moyo-R | Adults, 3days post emergence |
| Sample 16 | MS-L2 | Moyo-S | Larval instar 2 |
| Sample 17 | MS-L3 | Moyo-S | Larval instar 3 |
| Sample 18 | MS-L4-D1 | Moyo-S | Larval instar 4.day1 |
| Sample 19 | MS-L4-D2 | Moyo-S | Larval instar 4.day2 |
| Sample 20 | MS-Ad-3DPE | Moyo-S | Adults, 3days post emergence |
| Sample 21 | MR-N1 | Moyo-R | Naive blood feeding, 1day after feeding |
| Sample 22 | MR-N2 | Moyo-R | Naive blood feeding, 2 days after feeding |
| Sample 23 | MR-D1 | Moyo-R | Dengue virus mixed blood feeding, 1 day after feeding |
| Sample 24 | MR-D2 | Moyo-R | Dengue virus mixed blood feeding, 2 days after feeding |
| Sample 25 | MS-N1 | Moyo-S | Naive blood feeding, 1day after feeding |
| Sample 26 | MS-N2 | Moyo-S | Naive blood feeding, 2 days after feeding |
| Sample 27 | MS-D1 | Moyo-S | Dengue virus mixed blood feeding, 1 day after feeding |
| Sample 28 | MS-D2 | Moyo-S | Dengue virus mixed blood feeding, 2 days after feeding |
| Sample 29 | Antibiotic-treated-MR-3H-1 | Moyo-R | Antibiotic treatment.3 hours post blood feeding, sample 1 |
| Sample 30 | Antibiotic-treated-MR-3H-2 | Moyo-R | Antibiotic treatment.3 hours post blood feeding, sample 2 |
| Sample 31 | Antibiotic-treated-MR-3H-3 | Moyo-R | Antibiotic treatment.3 hours post blood feeding, sample 3 |
| Sample 32 | Antibiotic-treated-MS-3H-1 | Moyo-S | Antibiotic treatment.3 hours post blood feeding, sample 1 |
| Sample 33 | Antibiotic-treated-MS-3H-2 | Moyo-S | Antibiotic treatment.3 hours post blood feeding, sample 2 |
| Sample 34 | Antibiotic-treated-MS-3H-3 | Moyo-S | Antibiotic treatment.3 hours post blood feeding, sample 3 |
| Sample 35 | Antibiotic-untreated-MR-3H-1 | Moyo-R | No antibiotic treatment.3 hours post blood feeding, sample 1 |
| Sample 36 | Antibiotic-untreated-MR-3H-2 | Moyo-R | No antibiotic treatment.3 hours post blood feeding, sample 2 |
| Sample 37 | Antibiotic-untreated-MR-3H-3 | Moyo-R | No antibiotic treatment.3 hours post blood feeding, sample 3 |
| Sample 38 | Antibiotic-untreated-MS-3H-1 | Moyo-S | No antibiotic treatment.3 hours post blood feeding, sample 1 |
| Sample 39 | Antibiotic-untreated-MS-3H-2 | Moyo-S | No antibiotic treatment.3 hours post blood feeding, sample 2 |
| Sample 40 | Antibiotic-untreated-MS-3H-3 | Moyo-S | No antibiotic treatment.3 hours post blood feeding, sample 3 |
