# Supplementary material for: Multifaceted functional implications of an endogenously expressed tRNA fragment in the vector mosquito Aedes aegypti
Source: PLoS Negl Trop Dis. 2018 Jan 24;12(1):e0006186. doi: 10.1371/journal.pntd.0006186 (PMC5783352; doi:10.1371/journal.pntd.0006186)
Supplement: S2 Table — (DOCX) [file pntd.0006186.s002.docx]

**S2 Table**. List of 55 expressed tRFs with description of parent tRNAs. The mean and maximum read counts of tRFs are also listed.

| tRNA gene id | amino acid | anticodon | tRF class | tRF mean read count | tRF max read count |
| --- | --- | --- | --- | --- | --- |
| AAEL016390 | Glu | CTC | 5-Pre | 51.8 | 372 |
| AAEL016866 | Lys | TTT | 5-Pre | 27.3 | 308 |
| AAEL016867 | Ala | AGC | 5-Pre | 129.4 | 556 |
| AAEL016064 | Pro | CGG | 5tRF | 158.1 | 825 |
| AAEL016090 | Leu | GAG | 5tRF | 18.4 | 175 |
| AAEL016091 | His | ATG | 5tRF | 26.1 | 237 |
| AAEL016397 | Glu | CTC | 5tRF | 32.8 | 530 |
| AAEL016396 | Leu | AAG | 5tRF | 31.9 | 529 |
| AAEL016482 | Ala | AGC | 5tRF | 181.3 | 554 |
| AAEL016485 | Leu | GAG | 5tRF | 178.7 | 544 |
| AAEL016600 | Ala | TGC | D-loop | 37.3 | 239 |
| AAEL016777 | Cys | GCA | D-loop | 92 | 495 |
| AAEL016781 | Leu | CAG | D-loop | 94.8 | 501 |
| AAEL016900 | Met | CAT | D-loop | 189.6 | 752 |
| AAEL016902 | Met | CAT | D-loop | 926.5 | 6817 |
| AAEL016903 | Ala | AGC | D-loop | 304 | 817 |
| AAEL016904 | Met | CAT | D-loop | 297.8 | 804 |
| AAEL016934 | Ala | AGC | D-loop | 342.1 | 4275 |
| AAEL016933 | Val | AAC | D-loop | 315.1 | 3977 |
| AAEL016246 | Glu | CTC | A-loop | 38 | 545 |
| AAEL016244 | Asn | GTT | A-loop | 31 | 415 |
| AAEL016443 | Asp | GTC | A-loop | 39.1 | 267 |
| AAEL016589 | Val | AAC | A-loop | 17.3 | 210 |
| AAEL016713 | Ala | AGC | A-loop | 30.3 | 506 |
| AAEL016714 | Pro | TGG | A-loop | 33.4 | 523 |
| AAEL016761 | Ala | AGC | A-loop | 759.5 | 4602 |
| AAEL016763 | Ala | AGC | A-loop | 1368.8 | 8239 |
| AAEL016781 | Leu | CAG | A-loop | 224.5 | 720 |
| AAEL016782 | Ala | AGC | A-loop | 223.9 | 718 |
| AAEL016779 | Thr | TGT | A-loop | 249 | 846 |
| AAEL016930 | Glu | CTC | A-loop | 170.2 | 639 |
| AAEL016059 | Leu | TAA | T-loop | 17.3 | 126 |
| AAEL016061 | Arg | ACG | T-loop | 14 | 251 |
| AAEL016062 | Ser | CGA | T-loop | 29.8 | 269 |
| AAEL016284 | Asp | ATC | T-loop | 14 | 205 |
| AAEL016283 | Ala | AGC | T-loop | 14 | 205 |
| AAEL016300 | Ala | AGC | T-loop | 14.1 | 283 |
| AAEL016742 | Ala | AGC | T-loop | 217 | 1907 |
| AAEL016743 | Trp | CCA | T-loop | 204.3 | 1720 |
| AAEL016947 | Gln | CTG | T-loop | 37.2 | 266 |
| AAEL016948 | Ala | AGC | T-loop | 37.6 | 258 |
| AAEL016012 | Asp | GTC | 3-Pre | 205.4 | 580 |
| AAEL016015 | Gly | GCC | 3-Pre | 13456.3 | 52343 |
| AAEL016142 | Ala | AGC | 3-Pre | 169.2 | 990 |
| AAEL016291 | Ala | AGC | 3-Pre | 33.2 | 157 |
| AAEL016292 | Ala | AGC | 3-Pre | 33.6 | 158 |
| AAEL016343 | Ala | AGC | 3-Pre | 304.1 | 1161 |
| AAEL016346 | Ala | AGC | 3-Pre | 313.7 | 1155 |
| AAEL016542 | Phe | GAA | 3-Pre | 14 | 197 |
| AAEL016534 | Asp | GTC | 3-Pre | 29.6 | 355 |
| AAEL016533 | Gly | GCC | 3-Pre | 25.2 | 328 |
| AAEL016783 | Ala | AGC | 3-Pre | 1989 | 18595 |
| AAEL016785 | Ala | AGC | 3-Pre | 47 | 461 |
| AAEL016845 | Ala | AGC | 3-Pre | 2035.3 | 26685 |
| AAEL016846 | Ala | AGC | 3-Pre | 2060.4 | 26820 |
