# Supplementary figures and images for: Multifaceted functional implications of an endogenously expressed tRNA fragment in the vector mosquito Aedes aegypti
Source: PLoS Negl Trop Dis. 2018 Jan 24;12(1):e0006186. doi: 10.1371/journal.pntd.0006186 (PMC5783352; doi:10.1371/journal.pntd.0006186)

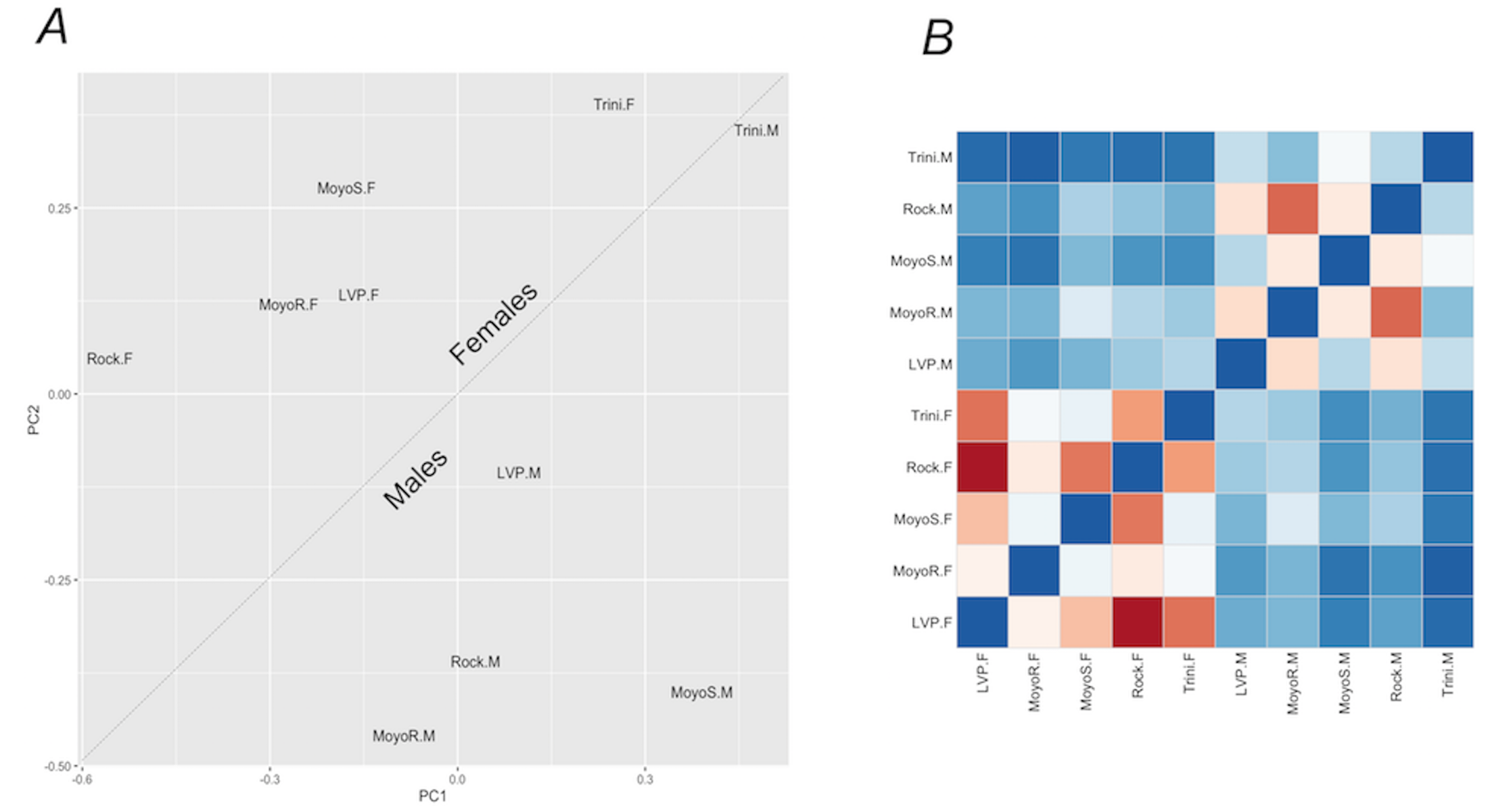

Supplement: S1 Fig — A) Principal component analysis of tRF abundance in males and females of 5 laboratory strains. The plot shows that males and females have different abundances of tRFs (the dotted line differentiates the two sexes). B) Plot of mutual information (MI) of variation of tRF abundance among males and females of the 5 strains. The pair-wise sample comparisons show MI value equals to 1 on the diagonal and the color code (blue to red) represents decreasing values of MI (MI value ranges from 0 to 1). (TIF) [file pntd.0006186.s003.tif]

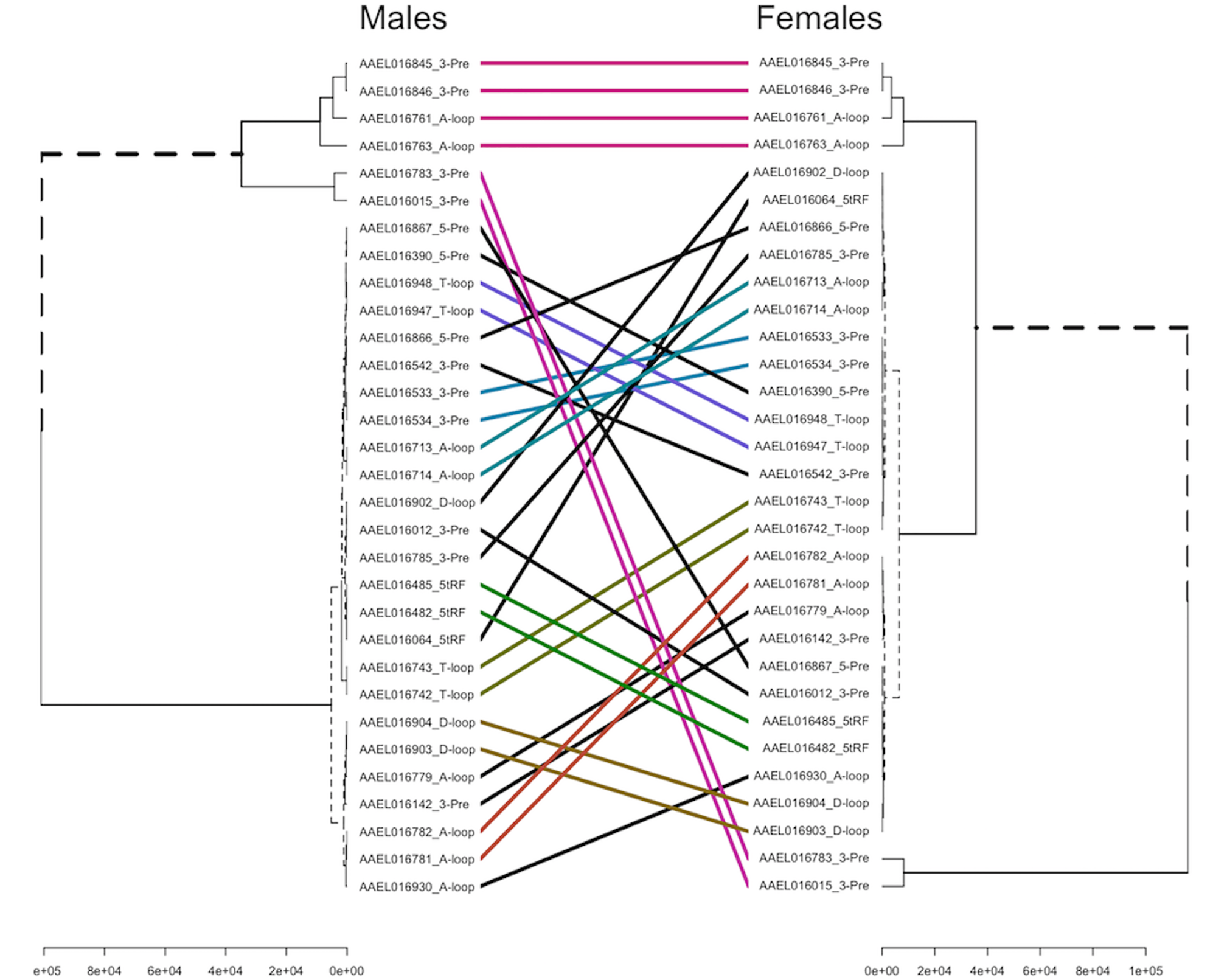

Supplement: S2 Fig — The variation of tRF expression in males across five strains is on left and that of females among the same strains are shown on right side of the plot. These expression patterns are shown as dendrograms of hierarchical clustering of tRF expression variation in males versus females. For example, the two tRFs AAEL016783_3-Pre and AAEL016015_3-Pre cluster together with AAEL016845_3-Pre, AAEL016846_3-Pre, AAEL016761_A-loop and AAEL016763_A-loop in males but cluster separately from those tRFs in females. The cluster branch and nodes are color coded and lines connecting the nodes between the two clusters indicate cluster position of tRFs in males vs. females. Dotted branches show samples with low cluster distance. The scales on the bottom represent branch lengths which were determined from calculating cluster distance by Ward’s method from expression data. (TIF) [file pntd.0006186.s004.tif]
